# Comparison of different bridging anticoagulation therapies used after mechanical heart valve replacement in Chinese patients - a prospective cohort study

**DOI:** 10.1186/s13019-020-1084-7

**Published:** 2020-02-24

**Authors:** Bo-Xia Li, Shi-Dong Liu, Liang Qi, Shusen Sun, Wei Sun, Yuan-Min Li, Bing Song, Xin-An Wu

**Affiliations:** 1 Department of Pharmacy, First Hospital of Lanzhou University, Lanzhou, 730000 China; 2 First Clinical Medical College, Lanzhou University, Lanzhou, 730000 China; 3 Cardiovascular Surgery, First Hospital of Lanzhou University, Lanzhou, 730000 China; 4 College of Pharmacy and Health Sciences, Western New England University, Springfield, MA 01119 USA

**Keywords:** Bridging anticoagulation, Mechanical heart valve replacement, Low-molecular-weight heparin, Chinese patients

## Abstract

**Objective:**

To assess different bridging anticoagulation therapies early after mechanical heart valve replacement (MHVR) in Chinese patients.

**Methods:**

We performed a prospective, single-center, observational cohort study of 305 patients who underwent elective MHVR with different bridging anticoagulation regimens. Patients enrolled in the study were divided into three bridging therapy groups: the unfractionated heparin (UFH) group (*n* = 109), the low-molecular-weight heparin (LMWH) group (*n* = 97), and the UFH with sequential LMWH (UFH-LMWH) group (*n* = 99). All patients were followed for 4 weeks.

**Results:**

Two patients experienced thromboembolic stroke events in the UFH group. The LMWH group was associated with an increase in the incidence of bleeding events compared with the UFH group (10.3% VS 2.8%; *P* = 0.03). With a comparison of LMWH and UFH group in secondary endpoints, the statistical test for significance indicated a trend of reduced ICU length of stay (*P* = 0.08), postoperative length of stay (P = 0.08) and time of achieving target INR (*P* = 0.06). The creatinine level (odds ratio = 1.03; 95% confidence interval = 1.01 to 1.05; *P* = 0.02) and hypertension (odds ratio = 3.72; 95% confidence interval = 1.35 to 10.28; *P* = 0.01) were risk factors for bleeding events.

**Conclusion:**

For Chinese patients, the LMWH bridging anticoagulation presents the increased the incidence of bleeding events, but enables patients to benefit from achieving an early anticoagulation effect. Close follow-up and personalized management are required in patients with thromboembolic and bleeding risk factors.

**Trial registration:**

Chinese Clinical Trial Registry ChiCTR1800019841. Registered 2 December 2018 retrospectively.

## Introduction

Bridging anticoagulation after mechanical heart valve replacement (MHVR) has been accepted as a standard of practice in cardiac surgery centers worldwide. However, how to manage bridging anticoagulation regimens remains a challenge with no existing accordance [1]. The American College of Chest Physician (ACCP) guidelines recommend the use of low-dose unfractionated heparin (UFH), low-dose low-molecular-weight heparin (LMWH) or therapeutic dose LMWH over therapeutic dose of UFH in 2012 [2]. Bridging anticoagulation with either intravenous UFH or subcutaneous LMWH is recommended in the 2014 American College of Cardiology/American Heart Association (ACC/AHA) guidelines. When LMWH is used, therapeutic weight-adjusted doses are given twice daily. The use of bridging heparin after surgery must be individualized, depending on the risks of bleeding and thrombosis [3]. The 2017 ESC/EACTS Guidelines for the Management of Valvar Heart Disease show that intravenous UFH monitored to an activated partial thromboplastin time (APTT) of 1.5–2.0 times the control value, enables rapid anticoagulation to be obtained before international normalized ratio (INR) rises [4]. Lack of accordance among the guidelines may generate a broad standard of practice in cardiovascular centers in bridging anticoagulation after MHVR [5].

The use of different bridging anticoagulation therapies after MHVR remains under discussion because of the risk of bleeding and thromboembolic events postoperatively [6]. Additionally, the doses of bridging anticoagulants and the target range of bridging anticoagulation are significantly different between Chinese cardiac surgery centers and foreign centers [7, 8]. At present, there is no clear Chinese guideline or consensus on bridging anticoagulation after MHVR, and few clinical studies have been conducted in Chinese patients [7–12].

We performed a prospective, single-center, observational cohort study to evaluate the efficacy and safety of different bridging anticoagulant regimens following early MHVR, and aim to provide Chinese evidence for the development of international guidelines.

## Patients and methods

### Study patients

From January 1, 2016, to December 1, 2018, 352 patients who underwent elective MHVR in the First Hospital of Lanzhou University were registered in the clinical trial before the surgery. The inclusion criteria were patient’s age ≥ 18 years and had MHVR surgery. The exclusion criteria were pregnancy, dialysis, aortic dissection, critical perioperative state, recent neurologic event and severe renal insufficiency (serum creatinine >150 μmol/L) before operation; bioprosthetic heart valve replacement, severe renal insufficiency (serum creatinine > 150 μmol/L), intra-aortic balloon counterpulsation, duration of intubation more than 48 h and patients who were administered LMWH for less than 2 days after surgery. Informed consent was obtained from all participants included in the study. The study was approved by the ethics committee of the First Hospital of Lanzhou University (LDYYLL2018–154).

### Study design

Patients who met the inclusion and exclusion criteria were recruited consecutively in the prospective, single-center, observational cohort study. The study was registered at the Chinese Clinical Trial Registry, registration number ChiCTR1800019841. During a surgical procedure, UFH was given to sustain an activated clotting time above 400 s. When the cardiopulmonary blood bypass was stopped, protamine sulfate was used to neutralize UFH anticoagulation. At the end of the surgery, two surgical drains were placed around the heart, and, if necessary, a third surgical drain was placed in the pleural cavities. In the early postoperative period, patients stayed in the ICU ward until they achieved respiratory and hemodynamic stability; then, they were transferred to the general ward. The surgical drains were removed when the drainage volume was less than 50 ml/d in the general ward.

Bridging anticoagulation was initiated at 6 h postoperatively either subcutaneous UFH 25 IU/kg/dose four times daily or subcutaneous LMWH 4000 IU of anti-Xa/dose twice daily. Warfarin, starting dose 3 mg, was given as soon as patients were extubated on the postoperative day 1 or 2. Bridging anticoagulants were given until INR was within the target range for 2 consecutive days (1.5 to 2.5 for aortic valve replacement, 1.8 to 3.0 for mitral valve replacement and bivalve replacement, 2.5 to 3.0 for tricuspid valve replacement) [13, 14].

**Fig. 1:**
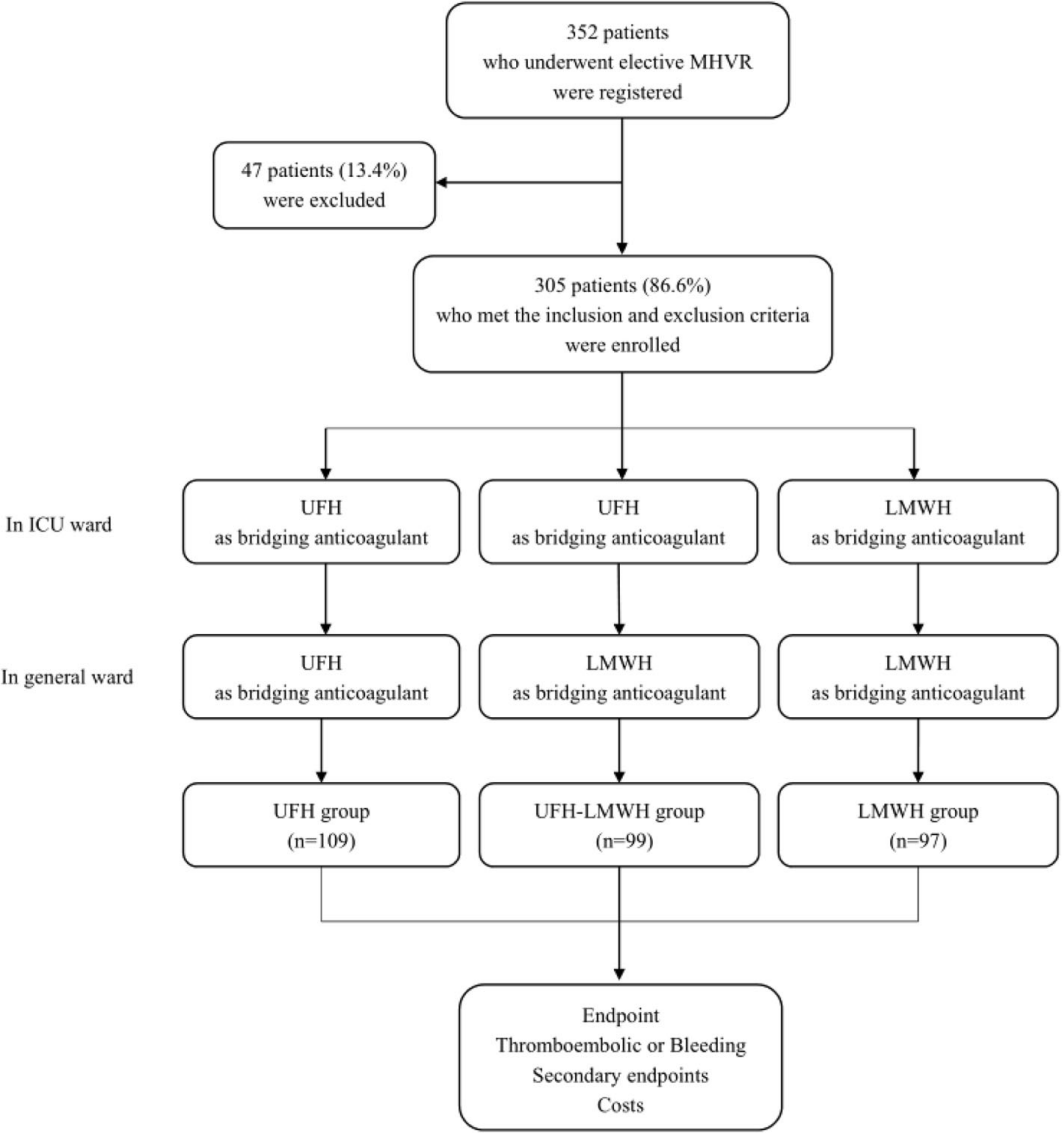
Study Flowchart

According to the physicians’ orders after surgery, patients enrolled in the study were divided into three groups: the UFH group, the UFH-LMWH group and the LMWH group. Regardless of ICU or general ward stays, UFH was used as a monotherapy in the UFH group, and LMWH was used as a monotherapy in the LMWH group. For patients in the UFH-LMWH group, UFH was used as monotherapy in the ICU, and it was replaced by LMWH in the general ward to bridge anticoagulation (Fig. 1). All patients were followed for 4 weeks after operation.

### Endpoints

The primary endpoint was the occurrence of thromboembolic or bleeding events during the 4-week follow-up. Thromboembolic events included transient stroke, permanent stroke, peripheral embolism, and valve thrombosis. Bleeding events included proved fatal bleeding, intracranial hemorrhage, retroperitoneal bleeding, requiring an intervention, transfusion of ≥2 U of red blood cells, resulting in chronic sequelae or prolongation of the hospital stay, epistaxis, airway bleeding, hematuria, hematemesis, gastrointestinal bleeding, and subcutaneous hemorrhage. The secondary endpoints included a volume of drainage, ICU length of stay, postoperative length of stay, and time of achieving target INR. The third endpoints were costs, including hospital costs, medicine costs and the drug share (ratio of medicine costs over hospital costs).

### Statistical analysis

Continuous variables are expressed as mean ± standard deviation (SD), and categorical variables are expressed as numbers (percentages). ANOVA adjusted by Bonferroni’s method was used to test for statistical significance in continuous data, and Chi-square test or Fisher’s exact test was used to determine statistical significance in categorical data. A *P* value of less than 0.05 was considered to be statistically different. Potential risk factors for thromboembolic or bleeding events were first tested by univariate analysis, and the variables tested included: gender, age, weight, body mass index (BMI), left ventricular ejection fraction (LVEF), CHA2DS2-VASc-Score, the New York Heart Association functional (NYHA) class, smoking status, hypertension, diabetes, atrial fibrillation, coronary artery disease, pulmonary hypertension, infective endocarditis, history of previous cardiovascular surgery, history of previous embolism, history of previous bleeding, hemoglobin level, platelet count, albumin, creatinine level, triglycerides, low density lipoprotein (LDL)-C, prothrombin time, fibrinogen level, operative characteristics, cross-clamp time, total bypass time, and postoperative bridging anticoagulation therapies.

Only significant variables with a *P* value less than 0.15 in the univariate analysis were used in a multivariable logistic regression analysis (with a forward, stepwise method based on the likelihood ratio test). The odds ratios and their corresponding 95% confidence intervals were showed in addition to their associated 2-sided *P*-values. All date were calculated and analyzed using Software Package for Statistics and Simulation (IBM SPSS version 22.0, IBM Corp Armonk, NY).

## Results

### Patient demographics and baseline characteristics

A totally of 352 patients who underwent elective MHVR were registered in the clinical trial before the surgery. Within this population, 305 patients (86.6%) met the inclusion and exclusion criteria, and received postoperative different bridging anticoagulation regimens. Thirty-one patients and 16 patients were excluded from cohort due to the preoperative and postoperative exclusion criteria, respectively. According to the postoperative physicians’ orders, patients enrolled in the study were divided into three bridging therapy groups: the UFH group (*n* = 109), the UFH-LMWH group (*n* = 99) and the LMWH group (*n* = 97). The patient demographics and baseline characteristics are shown in Table 1. Despite the lack of randomization, the three groups were well balanced for the patient demographics and baseline characteristics.

**Table 1:** Baseline Characteristics of Patients

Characteristic	the UFH group (*n* = 109)	the UFH-LMWH group (*n* = 99)	the LMWH group (*n* = 97)	*P* Value
Male, n (%)	63 (57.8%)	63 (63.6%)	51 (52.6%)	0.29
Age, years	50.3 ± 10.6	49.7 ± 11.1	52.4 ± 10.2	0.19
Weight, kg	63.1 ± 9.8	63.5 ± 10.3	64.4 ± 10.0	0.66
BMI, kg/m^2 a^	22.6 ± 3.0	22.3 ± 3.2	23.1 ± 2.7	0.17
LVEF, %	57.1 ± 5.8	55.7 ± 7.0	56.3 ± 6.4	0.23
CHA2DS2-VASc-Score	1.5 ± 0.9	1.5 ± 0.9	1.7 ± 1.0	0.12
NYHA^b^ class III or IV, n (%)	75 (68.8%)	65 (65.7%)	67 (69.1%)	0.85
Current smoker, n (%)	21 (19.3%)	24 (24.2%)	20 (20.6%)	0.67
Hypertension, n (%)	13 (11.9%)	16 (16.2%)	20 (20.6%)	0.24
Diabetes, n (%)	2 (1.8%)	3 (3.0%)	2 (2.1%)	0.83
Atrial fibrillation, n (%)^c^	34 (31.2%)	23 (28.9%)	32 (33.0%)	0.27
Coronary artery disease, n (%)	5 (4.6%)	8 (8.1%)	10 (10.3%)	0.29
Pulmonary hypertension, n (%)	17 (15.6%)	17 (17.2%)	15 (15.5%)	0.94
Infective endocarditis, n (%)	4 (3.7%)	4 (4.0%)	2 (2.1%)	0.71
History of aspirin, n (%)	4 (3.7%)	7 (7.1%)	8 (8.2%)	0.36
Previous cardiovascular surgery, n (%)	0 (0.0%)	1 (1.0%)	0 (0.0%)	0.35
Previous embolism, n (%)	5 (4.6%)	3 (3.0%)	4 (4.1%)	0.84
Previous bleeding, n (%)	3 (2.8%)	5 (5.1%)	3 (3.1%)	0.64
Biologic data				
Hemoglobin, g/L	143.2 ± 19.7	145.6 ± 23.0	141.1 ± 18.7	0.32
Platelet count, 10^9^/L	173.5 ± 69.0	171.4 ± 51.3	180.1 ± 66.0	0.60
Albumin, g/L	42.9 ± 3.7	42.4 ± 3.8	43.3 ± 3.2	0.20
Creatinine, μmol/L	71.4 ± 11.2	75.1 ± 20.7	76.7 ± 15.5	0.06
Triglycerides, mmol/L	1.2 ± 0.6	1.4 ± 0.9	1.3 ± 0.6	0.41
LDL-C, mmol/L^d^	2.5 ± 0.8	2.6 ± 0.9	2.6 ± 0.9	0.82
Prothrombin time, sec	12.7 ± 3.8	12.5 ± 3.5	12.4 ± 2.8	0.34
Fibrinogen, g/L	3.0 ± 0.8	2.9 ± 0.8	3.0 ± 0.9	0.76
Operative Characteristics				
AVR ^e^	33 (30.3%)	33 (33.3%)	29 (29.9%)	0.85
MVR ^f^	37 (33.9%)	25 (25.3%)	32 (33.0%)	0.34
BVR ^g^	22 (20.2%)	20 (20.2%)	21 (21.6%)	0.96
Bentall ^h^	17 (15.6%)	21 (21.2%)	15 (15.5%)	0.47
Cross-clamp time, minutes	82.8 ± 35.1	83.4 ± 36.1	82.6 ± 29.4	0.98
Total bypass time, minutes	121.6 ± 44.0	117.1 ± 43.0	117.0 ± 37.6	0.67

Continuous variables are expressed as mean ± SD; categorical variables are expressed as number (percentage)

^a^Body mass index (kg/m^2^); ^b^New York Heart Association functional class;

^c^Includes transient, persistent, permanent atrial fibrillation; ^d^Low density lipoprotein cholesterin

^e^Aortic valve replacement; ^f^Mitral valve replacement; ^g^Bivalve replacement

^h^Aortic valve replacement +ascending aorta replacement

### Primary endpoints

2(1.8%) patients who underwent MVRs experienced thromboembolic events at postoperative day 6 and 16, respectively, in the UFH group (Table 2). 1(0.9%) patient occurred permanent stroke at INR 1.77 during bridging anticoagulation; another 1(0.9%) patient occurred transient stroke at INR 2.15 during warfarin therapy alone. In the UFH-LMWH group and the LMWH group, none of the patients experienced thromboembolic event. The trial did not have enough thromboembolic events to provide evidence of treatment efficacy.

**Table 2:** Bleeding and Thromboembolic events

Endpoints events	the UFH group (*n* = 109)	INR ^a^	the UFH-LMWH group (*n* = 99)	INR	the LMWH group (*n* = 97)	INR
Bleeding events	3 (2.3%)	2.67	5 (6.3%)	2.69	10 (10.3%)	2.77
Epistaxis	1 (0.8%)	2.21	3 (3.8%)	2.31	7 (7.3%)	2.42
Airway bleeding	0 (0)	–	1 (1.3%)	4.05^b^	1 (1.0%)	3.85^b^
Hematuria	1 (0.8%)	2.38	0 (0)	–	1 (1.0%)	2.55
Hematemesis	0 (0)	–	1 (1.3%)	2.46	0 (0)	–
Gastrointestinal bleeding	1 (0.8%)	3.42^b^	0 (0)	–	1 (1.0%)	4.37^b^
Thromboembolic events	2 (1.6%)	1.96	0 (0)	–	0 (0)	–
Permanent stroke	1 (0.8%)	1.77	0 (0)	–	0 (0)	–
Transient stroke	1 (0.8%)	2.15	0 (0)	–	0 (0)	–

^a^The average INR value at the occurrence of the bleeding event

^b^The INRs were above the target range of the corresponding MHVR

**Fig. 2:**
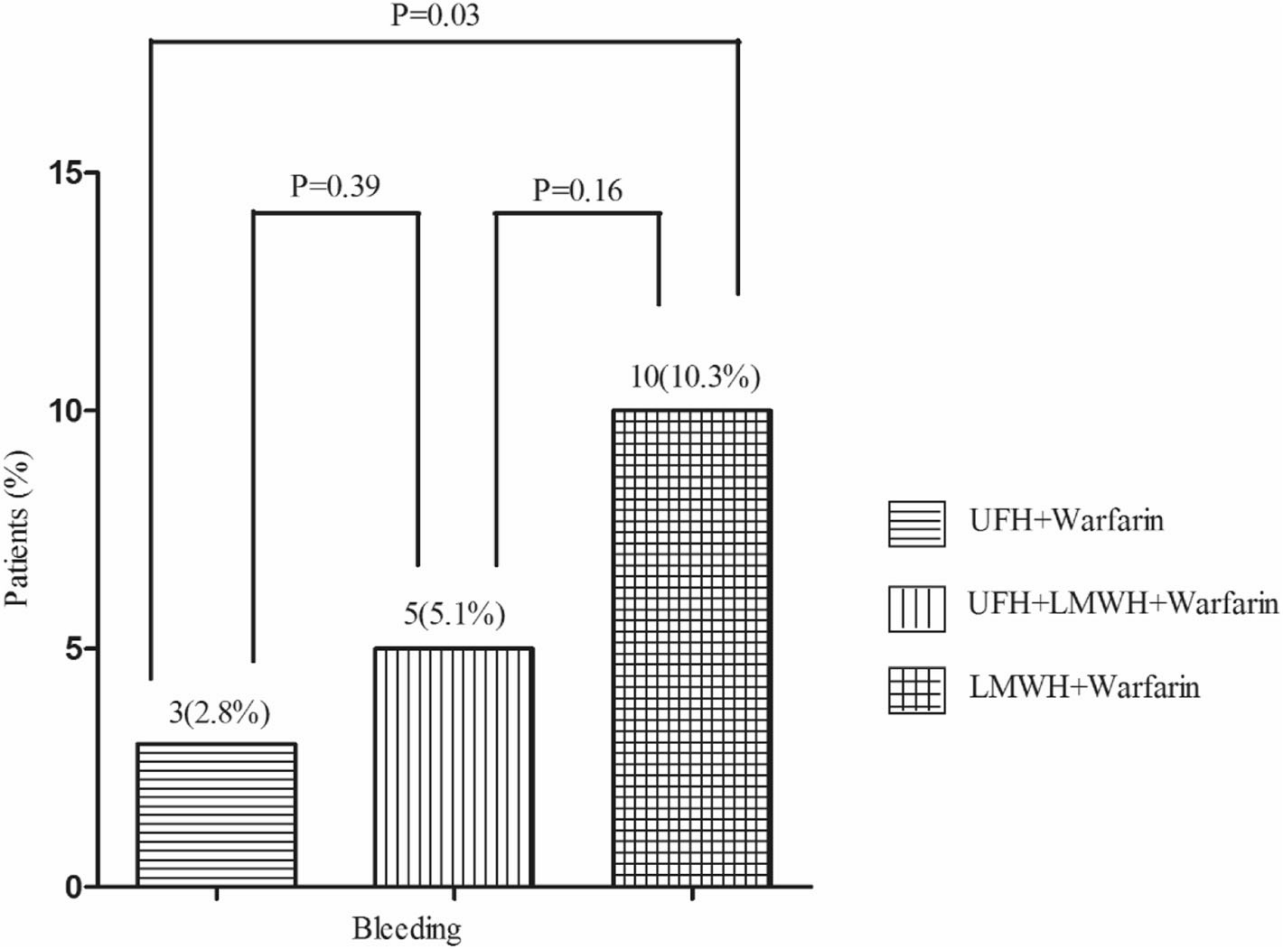
Incidence of bleeding events during 4 weeks between three groups

Bleeding events during 4 weeks of follow-up occurred in 3(2.8%) of patients in the UFH group, 5(5.1%) of patients in the UFH-LMWH group and 10(10.3%) of patients in the LMWH group (Table 3). These values represent a relative increase of 2.7 times in bleeding events with the LMWH group as compared with the UFH group (*P* = 0.03), indicating statistical significance. A relative increase of 82% in bleeding events in the UFH-LMWH group as compared with the UFH group (*P* = 0.39), and a relative increase of one time in bleeding events with the LMWH group as compared with the UFH-LMWH group (*P* = 0.16), both not meeting the criteria with statistical differences (Fig. 2).

**Table 3:** Endpoints

Variable	the UFH group (*n* = 109)	the UFH-LMWH group (*n* = 99)	*P* value	the LMWH group (*n* = 97)	*P* value
	Patients	Patients		Patients	
Primary endpoints					
All thromboembolic events	2 (1.8%)	0 (0)	–	0 (0)	–
thromboembolic events ^a^	1 (0.9%)	0 (0)	–	0 (0)	–
All bleeding events	3 (2.8%)	5 (5.1%)	0.39	10 (10.3%)	0.03
bleeding events ^b^	2 (1.8%)	3 (3.0%)	0.91	5 (5.2%)	0.35
Secondary endpoints					
Volume of drainage (ml)					
postoperative day 1	296.8 ± 186.2	291.2 ± 170.3	1.00	263.8 ± 175.3	0.55
postoperative day 2	203.5 ± 103.7	188.2 ± 113.2	0.95	187.5 ± 111.6	0.89
postoperative day 3	80.8 ± 66.9	91.5 ± 73.2	0.80	86.5 ± 68.4	1.00
postoperative day 4	40.0 ± 40.3	47.8 ± 49.6	0.68	40.8 ± 49.4	1.00
ICU length of stay (d)	4.2 ± 2.5	4.0 ± 1.4	0.97	3.7 ± 0.8	0.08
Postoperative length of stay (d)	15.8 ± 4.2	15.3 ± 3.5	0.98	14.6 ± 4.1	0.08
Time of achieving target INR (d)	10.9 ± 2.8	10.5 ± 3.2	0.78	10.0 ± 2.2	0.06
Third endpoints					
Hospital costs (yuan)	108,884.5 ± 26,641.7	109,900.5 ± 37,380.7	1.00	105,976.4 ± 22,249.5	1.00
Medicine costs (yuan)	41,214.3 ± 14,809.6	41,405.9 ± 13,368.3	1.00	39,176.9 ± 10,788.4	0.81
Medicine costs /Hospital costs	0.37 ± 0.07	0.38 ± 0.07	1.00	0.37 ± 0.07	1.00

Continuous variables are expressed as mean ± SD; categorical variables are expressed as number (percentage

^a^Thromboembolic events occurred during bridging anticoagulation

^b^Bleeding events occurred during bridging anticoagulation

All bleeding events were minor bleeding events, as defined by International Society of Thrombosis and Haemostasis (ISTH). The INRs were above the target range in 4(22.2%) and the INRs of other 14(77.8%) were below or within the target range, all of whom presented with a bleeding event (Table 2). Within the period of bridging anticoagulation, 2(1.8%) patients in the UFH group, 3(3.0%) patients in the UFH-LMWH group, 5(5.2%) patients in the LMWH group occurred bleeding events, but no statistical difference was found among the three groups (Table 3).

### The secondary endpoints

**Fig. 3:**
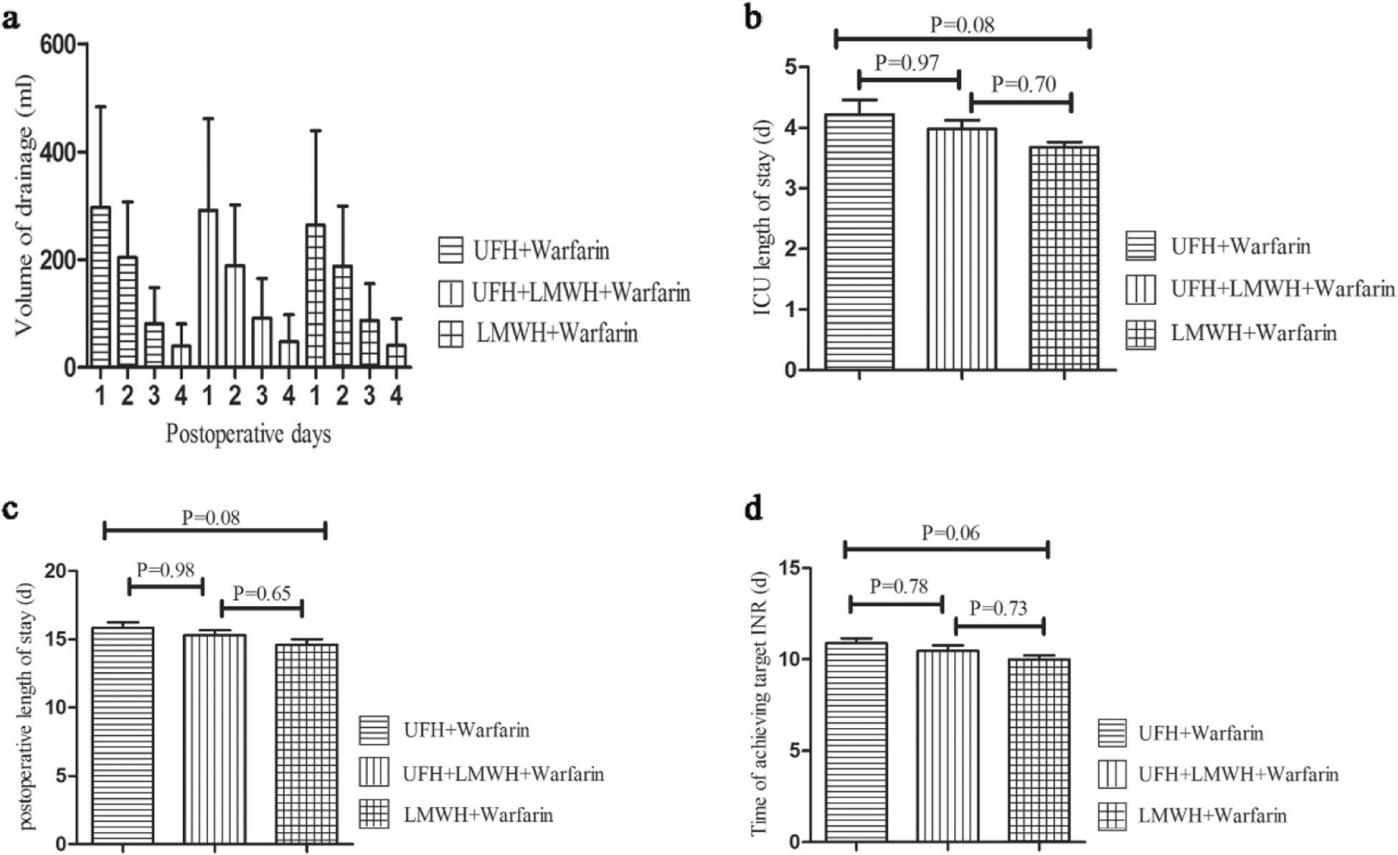
The secondary endpoints between three groups including (**a**) volume of drainage 4 days after surgery, (**b**) ICU length of stay, (**c**) postoperative length of stay, (**d**) time of achieving target INR

With a comparison of LMWH and UFH group in secondary endpoints, volume of drainage 4 days after surgery, ICU length of stay (3.7 ± 0.8 VS 4.2 ± 2.5; *P* = 0.08), postoperative length of stay (14.6 ± 4.1 VS 15.8 ± 4.2; *P* = 0.08), and time of achieving target INR (10.0 ± 2.2 VS 10.9 ± 2.8; *P* = 0.06) were not statistically different. The secondary endpoints had no statistical differences with the UFH-LMWH group as compared with the UFH group in volume of drainage 4 days after surgery, ICU length of stay, postoperative length of stay and time of achieving target INR (Table 3). Similarly compared with the UFH-LMWH group, the LMWH group also had no statistical differences in secondary endpoints (Fig. 3).

### The third endpoints

There were no statistically differences in hospital costs, medicine costs and the drug share among the three groups (Table 3).

### Analysis of risk factors for bleeding

**Fig. 4:**
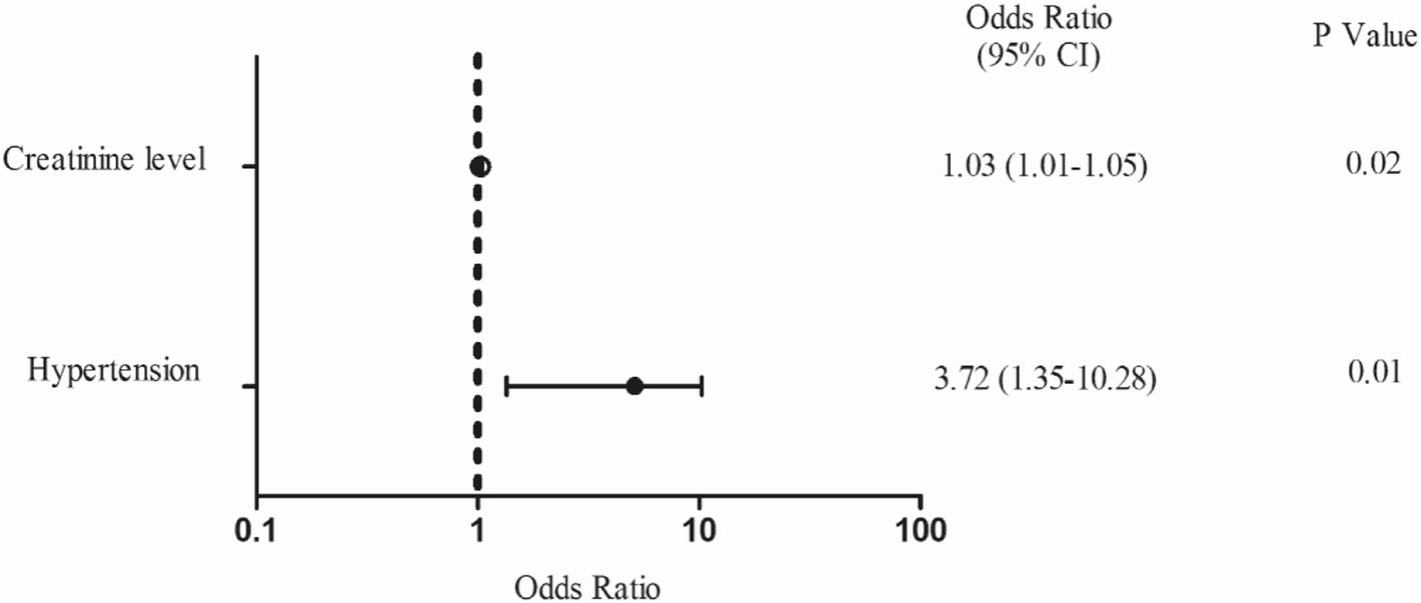
Risk factors of bleeding events in patients after MHVR based on a multivariate analysis

The trial did not have enough thromboembolic events to use a multivariable logistic regression analysis. Risk factors for bleeding events identified by univariate analysis were: male gender (56.8% VS 77.8% in control and bleeding event groups, respectively; *P* = 0.13); weight (63.4 ± 9.9 kg VS 67.7 ± 11.4 kg in control and bleeding event groups, respectively; *P* = 0.08); CHA_2_DS_2_-VASc score (1.56 ± 0.94 VS 1.94 ± 0.87 in control and bleeding event groups, respectively; *P* = 0.09); the New York Heart Association class III or greater (66.6% VS 88.9% in control and bleeding event groups, respectively; *P* = 0.09), hypertension (14.6% VS 38.9% in control and bleeding event groups, respectively; *P* = 0.01), creatinine level (73.7 ± 15.6 μmol/L VS 83.0 ± 22.8 μmol/L in control and bleeding event groups, respectively; *P* = 0.11); and postoperative bridging anticoagulation therapy (*P* = 0.07). The multivariable logistic regression analysis revealed that the creatinine level (odds ratio = 1.03; 95% confidence interval = 1.01 to 1.05; *P* = 0.02) and hypertension (odds ratio = 3.72; 95% confidence interval = 1.35 to 10.28; *P* = 0.01) were risk factors for bleeding events (Fig. 4).

## Discussion

Our prospective, single-center, observational cohort study demonstrated that using LMWH monotherapy increased the incidence of bleeding events after elective MHVR, but contributed to a significant reduction in ICU length of stay. Additionally, this study revealed that the creatinine level and hypertension were risk factors of bleeding events.

Regarding bridging anticoagulation protocol, subcutaneous UFH or LMWH bridging anticoagulant was administered 6 h after surgery, and warfarin was administered postoperative day 1 or 2 after extubation. Patients’ INRs were reviewed intermittently during hospitalization, and warfarin doses were adjusted according to INRs in order to reach target INRs stably. When INRs were stable for more than 2 days, UFH and LMWH were discontinued. The therapeutic range of INR for aortic or mitral valve replacement differs from the values recommended by the European or North American Societies (EACTS/ESC and AHA/AATS), however, Haibo Z’s and Dong L’s studies proved that the relatively low anticoagulant strategy efficiently prevents thrombosis and hemorrhage complications in the Chinese patients [13.14].

In our prospective, observational cohort study, all patients enrolled in the study were divided into three groups according to the postoperative physicians’ orders without randomization. On the other hand, rigorously screened and excluded patients based on inclusion and exclusion criteria. These generated unequal patient numbers among the three study cohorts. Nevertheless, the various comorbidities, which might bear an additional risk for thromboembolism or bleeding complications, were not significantly different among three groups.

The three study cohorts had comparable CHA_2_DS_2_ -VASc score, which provides a way to evaluate the difference in thromboembolic risk before MHVR. Two patients suffered a thromboembolic stroke at 6 and 16 days after surgery, respectively, in the UFH group. Both patients had atrial fibrillation and pulmonary hypertension, and one of whom had infective endocarditis and history of embolism. Although the trial did not have enough thromboembolic events to provide evidence of treatment efficacy, the occurrence of thromboembolic stroke demonstrates the necessity of bridging anticoagulation and personalized management. The dose of warfarin can be appropriately increased to prevent thromboembolic events for patients with high-risk factors for embolism.

Our findings, in terms of bleeding event rates in different bridging anticoagulation therapies following elective MHVR, were similar to published studies. In previous studies [7.9–12], the incidence of bleeding events in the UFH group were 1.8 to 10%, and the incidence of bleeding events in the LMWH group were 0.8 to 10%. Although the rate of bleeding events in the LMWH group (9.3%) was higher than that of the other two groups (2.3% or 6.3%) in our study, no statistical difference was found in the incidence of bleeding events during bridging anticoagulation. LMWH has a longer elimination half-life compared to heparin [15] and vitamin K and protamine sulfate are antagonists for warfarin and heparin [16]. This means that using LMWH bridging anticoagulation is a huge challenge for postoperative bleeding events.

Besides, compared with foreign cardiac surgery centers, the postoperative bridging anticoagulant dose (either therapeutic or prophylactic) was lower in Chinese cardiac surgery centers. In our study, the dose of UFH (25 IU/kg per dose four times daily) is lower than the prophylactic dose and the dose of LMWH (4000 IU of anti-Xa per dose twice daily) is between the therapeutic dose and the prophylactic dose used in foreign countries [7, 8]. In 18 patients presented with bleeding events, 4 (22.2%) patients’ INRs were above the target range, and the INRs of other 14 (77.8%) were in the target range. These can reflect the racial corporeity and high sensitivity of Chinese patients to anticoagulation. So a first prospective cohort study was performed to assess different bridging anticoagulation therapies used early after MHVR in Chinese patients and provide Chinese evidences for the development of related guidelines or consensus.

Notably, mainly, the relevant data and medical costs related to bridging therapy were collected, and these data, in general, were not reported in previous studies. The statistical test for significance indicated a shortening trend, although significance is missed in ICU length of stay (*P* = 0.08), postoperative length of stay (*P* = 0.08), and time of achieving target INR (*P* = 0.06), which has been reported in previous studies [11]. Despite 10 bleeding events in the LMWH group, all bleeding events were minor bleeding events, which can hardly delay the ICU length of stay or increase volume of drainage. On the other hand, previous studies [17] have shown that heparin and LMWH appear to be a dose-dependent safe, and effective anti-inflammatory agent. Compared with the other two groups, the bridging anticoagulant dose in the LMWH group was the highest, which may result in a shortening trend in ICU length of stay. Additionally, the reduced time of achieving target INR enables patients to benefit from the anticoagulation effect of warfarin earlier, and the prior discharge can lower hospital costs and medicine costs. Higher-level studies with larger sample sizes, longer follow-up, or randomized prospected controlled trial are needed to explore whether LMWH can shorten the ICU length of stay, postoperative time, and time of achieving target INR.

Meanwhile, the creatinine level and hypertension were identified as two bleeding risk factors through univariate analysis. The two factors were included in the items of HAS-BLED score, which was initially proposed to assess the 1-year bleeding risk of patients with atrial fibrillation and oral anticoagulation therapy [18]. This represents a “real world” about bleeding risk factors after implantation of a mechanical heart valve and can demonstrate that close follow-up and personalized management were required in patients with bleeding risk factors.

### Limitations

Nonrandomization is the main limitation of the prospective study. Posteriorly, lack of enough thromboembolic events to evaluate the efficacy of bridging anticoagulation, but the occurrence of permanent thromboembolic stroke demonstrates the necessity of bridging anticoagulation and personalized management. Moreover the endpoint lacked an assessment for early postoperative mortality, which was related to strict exclusion criteria that precluded patients with critical perioperative states. Further studies with larger sample sizes, longer follow-up or randomized prospected controlled trial are needed to confirm our findings.

## Conclusions

For Chinese patients, LMWH bridging anticoagulation exists challenge of increasing the incidence of bleeding events, but exists a trend that enables patients to benefit from the anticoagulation effect earlier. Close follow-up and personalized management are required in patients with thromboembolic and bleeding risk factors.

## Data Availability

The dataset analyzed during the current study may be available from the authors on reasonable request.

## Abbreviations

ACC/AHA: American College of Cardiology/American Heart Association
ACCP: American College of Chest Physician
APTT: Activated partial thromboplastin time
BMI: Body mass index
ICU: Intensive Care Unit
INR: International normalized ratio
ISTH: International Society of Thrombosis and Haemostasis
LDL: Low-density lipoprotein
LMWH: Low-molecular-weight heparin
LVEF: Left ventricular ejection fraction
MHVR: Mechanical heart valve replacement
NYHA: New York Heart Association
UFH: Unfractionated heparin
